# Visual symptom burden after traumatic brain injury: a case-control evaluation of the Arabic BIVSS

**DOI:** 10.3389/fnins.2026.1827721

**Published:** 2026-05-28

**Authors:** Nawaf M. Almutairi

**Affiliations:** Department of Optometry, College of Applied Medical Sciences, Qassim University, Buraydah, Saudi Arabia

**Keywords:** BIVSS, concussion, neuro-ophthalmology, neuro-optometry, symptom assessment, traumatic brain injury, vision disorders, visual rehabilitation

## Abstract

**Purpose:**

To compare visual symptoms between adults with traumatic brain injury (TBI) and controls using the Brain Injury Vision Symptom Survey (BIVSS) and identify the domains with the largest differences.

**Methods:**

In this prospective cross-sectional case–control study, 43 adults with TBI and 54 controls in Qassim, Saudi Arabia, completed the 28-item BIVSS. Total and eight domain scores (visual clarity, visual comfort, double vision, light sensitivity, dry eye, depth perception, peripheral vision, reading) were compared with Welch’s *t*-tests and Cohen’s d; domain-level analyses were exploratory and uncorrected for multiple comparisons. Discrimination of the total score was assessed by unadjusted and age- and gender-adjusted logistic regression and by exploratory ROC analysis; cut-off values are reported as sample-specific estimates. An exploratory subgroup analysis compared acute (≤30 days; *n* = 9) and chronic (>30 days; *n* = 34) TBI cohorts cross-sectionally.

**Results:**

Groups were similar in age and gender. TBI participants reported higher total BIVSS scores than controls (27.9 vs. 13.1; *p* < 0.001; *d* = 1.15) and higher scores across all eight domains (all *p* ≤ 0.016, uncorrected; *d* = 0.52–1.11), with the largest differences in visual comfort (*d* = 1.11) and reading (*d* = 0.81). Each 1-point increase was associated with higher odds of TBI (OR 1.106, 95% CI 1.057–1.157); the association was essentially unchanged after adjustment for age and gender. The total score showed moderate-to-good discrimination (AUC = 0.79). In an exploratory cross-sectional subgroup analysis, the acute group (*n* = 9) had higher overall scores than the chronic group (36.3 vs. 25.7), with a large cross-sectional difference in depth perception (*d* = 1.93 acute vs. control; *d* = 0.46 chronic vs. control); these acute–chronic differences are hypothesis-generating and cannot establish recovery.

**Conclusion:**

Adults with TBI reported substantially greater vision-related symptoms on the Arabic BIVSS than controls, with prominent differences in two out of eight domains: visual comfort and reading. The BIVSS supports symptom profiling to flag patients for targeted visual assessment after TBI; it is not a diagnostic test, and cut-offs are exploratory and sample-specific, requiring external validation. Cross-sectional differences between acute and chronic subgroups warrant longitudinal study before inferences about recovery can be drawn.

## Introduction

Traumatic brain injury (TBI) is a major global health problem and a leading cause of long-term disability, with substantial societal and healthcare burden (Dewan et al., 2018; James et al., 2019; Maas et al., 2017). TBI is defined as an alteration in brain function, or other evidence of brain pathology, caused by an external force (Menon et al., 2010). Beyond the initial mechanical insult, TBI triggers evolving neurometabolic and neuroinflammatory processes that may persist and contribute to chronic symptomatology, even after mild injury (mTBI) or concussion (Giza and Hovda, 2014; Patterson and Holahan, 2012). The neurometabolic cascade involves ionic imbalances, energy crisis, and axonal dysfunction that can impair cerebral function for days to weeks following injury (Giza and Hovda, 2014), while the neuroinflammatory response characterized by microglial activation and cytokine release may persist for months and has been suggested to be more damaging than the initial impact itself (Patterson and Holahan, 2012).

Visual dysfunction is increasingly recognized as a common and clinically meaningful consequence of TBI (Armstrong, 2018; Ciuffreda et al., 2007; Merezhinskaya et al., 2019). This vulnerability reflects the distributed organization of visual sensory processing and oculomotor control across cortical, cerebellar, brainstem, and vestibular pathways, such that diffuse injury or network dysfunction can disrupt efficient visual performance (Armstrong, 2018; Felleman and Van Essen, 1991; Goodrich et al., 2013). Across civilian and military cohorts, common findings include accommodative dysfunction, vergence disorders (notably convergence insufficiency), and oculomotor abnormalities affecting saccades and smooth pursuit (Alvarez et al., 2012; Ciuffreda et al., 2007; Felleman and Van Essen, 1991; Goodrich et al., 2013; Hellerstein et al., 1995; Master et al., 2016; Merezhinskaya et al., 2019). In a systematic review and meta-analysis of patients with TBI without direct ocular injury, pooled prevalence estimates were high for accommodative dysfunction and convergence insufficiency, with additional burden from visual field loss (Merezhinskaya et al., 2019). These deficits frequently manifest as blurred vision, diplopia, visual fatigue, photophobia, and reading difficulty symptoms that can be disproportionate to routine neurological findings (Armstrong, 2018; Ciuffreda et al., 2007; Digre and Brennan, 2012; Hellerstein et al., 1995).

Importantly, visual symptoms may be under-identified in routine post-TBI care, despite their potential to compound headaches, dizziness, and cognitive complaints and to interfere with academic/work performance and rehabilitation participation (Armstrong, 2018; O’Neil et al., 2013). Evidence syntheses emphasize variability in assessment approaches and the need for more standardized screening and evaluation pathways that align patient-reported symptoms with targeted clinical testing (O’Neil et al., 2013; Ventura et al., 2016). In concussion care, structured ocular/vestibular screening approaches (e.g., vestibular/ocular motor screening) and oculomotor-based measures have been proposed to improve detection of clinically relevant dysfunction (Mucha et al., 2014; Ventura et al., 2016).

Because post-TBI visual dysfunction is common and can meaningfully affect function, yet routine pathways for identifying and addressing these problems remain variable, a standardized symptom measure can help clinicians recognize patients who warrant targeted vision assessment and referral (Merezhinskaya et al., 2019; O’Neil et al., 2014). The BIVSS is a 28-item, TBI-oriented self-report survey designed to quantify vision-related symptom burden; importantly, it is a screening and symptom-profiling tool rather than a diagnostic test for specific underlying binocular, accommodative, or oculomotor disorders (Laukkanen et al., 2017).

Region-specific data are also important because mechanisms of injury, pathways to rehabilitation, and access to specialized services vary internationally. In the Middle East, TBI epidemiology and outcomes are heterogeneous, and road traffic injuries are a major contributor in several settings (Al-Hajj et al., 2021; El-Menyar et al., 2017). In Saudi Arabia specifically, published head-injury epidemiology and rehabilitation cohorts underscore a substantial burden and the importance of optimizing post-acute services and functional recovery pathways (Al-Habib et al., 2013; Almutairi et al., 2025a). However, prospective studies that quantify post-TBI visual symptoms using validated, culturally adapted symptom surveys in Saudi populations remain limited, supporting the need for locally generated evidence to guide clinical pathways and referral decisions (Al-Hajj et al., 2021; Almutairi et al., 2025a).

Therefore, the objective of this study was to evaluate the frequency and severity of vision-related symptoms in adults with TBI in the Qassim region of Saudi Arabia using an Arabic version of the BIVSS and to compare symptom profiles with those of a non-injured control group. We further aimed to assess the discriminative validity of Arabic BIVSS total and domain scores for identifying TBI-associated visual symptom burden in this setting.

## Materials and methods

### Study design and setting

We conducted a prospective, cross-sectional study at clinical sites affiliated with hospitals in the Qassim region, Saudi Arabia. The study was approved by the Committee of Research Ethics at Qassim University and adhered to the Declaration of Helsinki. All participants provided written informed consent.

### Participants

Participants were recruited by convenience sampling from neurology, rehabilitation, and general outpatient clinics. The TBI group comprised adults (≥18 years) with a clinical history of mild to severe TBI. Time since injury was not restricted; both acute and chronic cases were included. A subset of the TBI cohort (*n* = 29) also completed a 1-month retest as part of a separate cross-cultural adaptation and test–retest reliability evaluation of the Arabic BIVSS. The present case–control analysis is distinct in aim and focuses on between-group differences, discriminative validity, and ROC-based threshold exploration using a control group. Exclusion criteria for the TBI group were any significant ocular pathology unrelated to TBI (e.g., advanced glaucoma, macular degeneration), severe cognitive impairment preventing independent questionnaire completion, active psychiatric illness, or inability to provide informed consent. The control group comprised adults without a history of TBI, recruited from the student body of Qassim University and screened to exclude major ocular disease or neurological disorders affecting vision. Demographic information (age, gender) was recorded for all participants. For TBI participants, duration of injury (days since event) and documented severity were recorded.

### Measure: Brain Injury Vision Symptom Survey (BIVSS)

All participants completed the BIVSS in Arabic, with a researcher on hand to clarify any items if needed. The Arabic version used in this study was developed through standardized cross-cultural adaptation and evaluated for reliability in a companion validation study (Almutairi et al., 2026). The BIVSS consisted of 28 items scored on a 5-point Likert scale from 0 (“never”) to 4 (“always”) (Laukkanen et al., 2017). Item scores were summed to form a total score (range 0–112). In addition, we computed eight domain scores: visual clarity, visual comfort, double vision, light sensitivity, dry eye, depth perception, peripheral vision, and reading, by summing the relevant items for each domain. The present analysis is a clinical case–control comparison of Arabic BIVSS scores. Full psychometric validation of the Arabic version, including item-level analysis, internal consistency, and test–retest reliability, is reported in the companion paper (Almutairi et al., 2026). Cross-language measurement invariance, differential item functioning, and clinically validated screening thresholds were not assessed in this study, and no such claims are made for the Arabic version on the basis of these data.

### Statistical analysis

Analyses were performed using standard statistical software. Age and gender were compared between groups using Welch’s *t*-test and chi-square test, respectively. For the BIVSS, we calculated descriptive statistics (mean, SD, median, range) for total and domain scores by group.

Between-group differences in total and domain scores were examined using Welch’s *t*-tests (allowing for unequal variances). Effect sizes were quantified with Cohen’s *d* (difference in means divided by pooled standard deviation). For the total score, the following was conducted:

Generated a ROC curve and calculated the area under the curve (AUC).

Calculated sensitivity and specificity at different cut-off scores.

Ran two logistic regression models with group (TBI vs. control) as the dependent variable: an unadjusted model with BIVSS total score as the sole predictor, and a model adjusted for age and gender. Odds ratios with 95% confidence intervals are reported for both models.

The case–control design is well suited to estimating discrimination (AUC) but yields prevalence-dependent positive and negative predictive values that reflect the case–control sampling ratio rather than the true clinical prevalence of TBI-related visual symptoms; cut-off, PPV, and NPV values are therefore presented as exploratory, sample-specific estimates only and require external validation in independent prospective samples.

A two-sided *p* < 0.05 was considered statistically significant. Because this paper focuses on clinical interpretation, we emphasize effect sizes and patterns across domains, rather than formal correction for multiple comparisons.

## Results

### Participant characteristics

A total of 97 participants were included: 43 in the TBI group and 54 in the control group. The TBI group had a mean age of 27 ± 11.5 years (range 18–63), and the control group had a mean age of 25.0 ± 6.8 years (range 18–55); this difference was not statistically significant (*p* = 0.305). The TBI group comprised 27 females (62.8%) and 16 males (37.2%), whereas the control group comprised 24 females (44.4%) and 30 males (55.6%) (χ^2^ = 2.54, *p* = 0.111) (Table 1).

**TABLE 1:** Demographic and clinical characteristics of the study participants.

Characteristic	TBI group (*n* = 43)	Control group (*n* = 54)	*P*-value
Age (years)			
Mean ± SD	27.0 ± 11.5	25.0 ± 6.76	0.305
Range	18–63	18–55	
Gender, *n* (%)			0.111
Female	27 (62.8%)	24 (44.4%)	
Male	16 (37.2%)	30 (55.6%)	
Injury characteristics (TBI only)			
Median time since injury	730 days (∼2.0 years)	N/A	
IQR (days)	122–3650	N/A	
Range (days)	1–9490	N/A	
Injury severity/presentation (n = 33)			
Mild	11	N/A	
Moderate	10	N/A	
Severe	3	N/A	
Emergency/acute*	9	N/A	

*Recruited in the emergency department without a formal Glasgow Coma Scale category. Values are presented as mean ± SD for age and frequency (%) for gender.

Among TBI participants, the median time since injury was 730 days (interquartile range 122–3650; range 1–9490 days). Severity/presentation classification was available for 33 of 43 cases: 11 were recorded as mild, 10 as moderate, 3 as severe, and 9 were recruited in the emergency department without a formal Glasgow Coma Scale category; severity was missing for 10 cases. No participants in either group had major ocular pathology that met the exclusion criteria.

### BIVSS total score and discriminative validity

Traumatic brain injury participants had markedly higher BIVSS total scores than controls. The TBI group mean was 27.9 ± 16.6 (median 29; range 2–75) compared with 13.1 ± 9.0 (median 12.5; range 0–34) in controls. This difference was statistically significant (Welch’s *t* = 5.29, *p* < 0.001) with a very large effect size (Cohen’s *d* = 1.15). Results were essentially unchanged in a logistic regression model adjusted for age and gender, with the BIVSS total score remaining strongly associated with TBI status.

In the unadjusted logistic regression, each 1-point increase in BIVSS total score was associated with approximately 10.6% higher odds of being in the TBI group (OR 1.106, 95% CI 1.057–1.157, *p* < 0.001). A 10-point increase corresponded to an approximately 2.7-fold increase in odds of TBI (OR 2.73, 95% CI 1.74–4.30).

**FIGURE 1:**
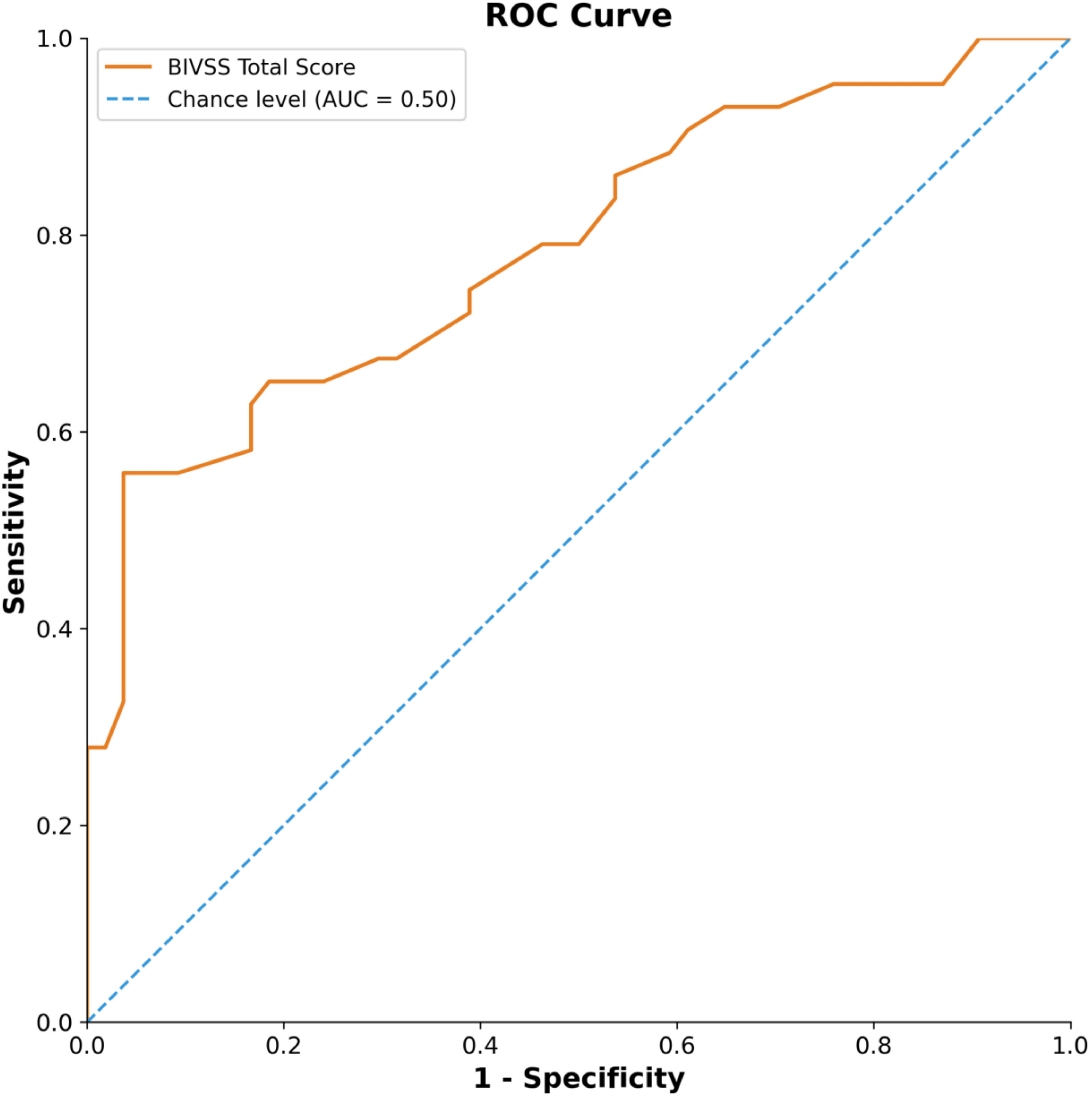
Receiver operating characteristic (ROC) curve for the BIVSS total score.

Receiver operating characteristic (ROC) analysis in this case–control sample yielded an area under the curve (AUC) of 0.79 (Figure 1), consistent with moderate discrimination that requires external validation in independent samples. As an exploratory exercise, candidate cut-off values were derived from the same case–control sample without external validation; these are reported as sample-specific anchor points only and should not be interpreted as clinically validated screening thresholds. Using Youden’s J index, the sample-specific maximum-Youden cut-off was ≥28 points, yielding a low sensitivity of 55.8% and high specificity of 96.3%. Alternatively, a cutoff of ≥22 points provided a more balanced trade-off with sensitivity of 65.1% and specificity of 81.5% (Supplementary Appendix Table 1). Because the present study used a case–control sampling design with a TBI prevalence of approximately 44% by construction, the positive and negative predictive values reported in Supplementary Appendix Table 1 are not directly generalizable to clinical populations with different prevalence; they are presented as illustrative, sample-specific values only.

### Domain-level differences

Symptom scores were significantly higher in the TBI group across all 8 BIVSS domains (all *p* ≤ 0.016, uncorrected), with effect sizes ranging from moderate to large (*d* = 0.52–1.11). The largest between-group differences were observed for Visual Comfort (*d* = 1.11) and Reading (*d* = 0.81), highlighting these as key areas of symptom burden in the TBI cohort (Table 2). Because eight domain comparisons were performed without formal correction for multiple testing, these domain-level *p*-values are exploratory and the pattern of effect sizes should be interpreted as descriptive rather than confirmatory.

**TABLE 2:** Brain Injury Vision Symptom Survey (BIVSS) domains-level symptoms comparison between TBI and the control participants.

Domain	TBI (*n* = 43)	Control (*n* = 54)	*P*-value	Cohen’s d
Visual clarity	3.9 ± 4.3	1.9 ± 2.1	0.006	0.63
Visual comfort	6.6 ± 4.3	2.6 ± 2.8	<0.001	1.11
Double vision	2.3 ± 2.3	1.4 ± 1.6	0.016	0.52
Light sensitivity	3.3 ± 3.3	1.7 ± 1.9	0.008	0.59
Dry eye	4.3 ± 2.9	2.9 ± 2.5	0.012	0.54
Depth perception (stereopsis)	1.4 ± 2.3	0.3 ± 1.1	0.005	0.65
Peripheral vision	1.8 ± 2.2	0.7 ± 1.2	0.005	0.64
Reading	4.2 ± 4.1	1.5 ± 2.6	<0.001	0.81

### Subgroup analysis: acute vs. chronic TBI

To explore potential differences in symptom presentation over time, we performed an exploratory stratification of the TBI group into acute (≤30 days post-injury; *n* = 9) and chronic (>30 days post-injury; *n* = 34) subgroups. The acute subgroup had a mean time since injury of 11.4 ± 10.8 days, while the chronic subgroup had a median duration of 1278 days (approximately 3.5 years). Demographic and clinical characteristics of these subgroups are presented in Supplementary Appendix Table 2. Age did not differ significantly across subgroups (*p* = 0.059), whereas gender distribution did (*p* = 0.042; the acute subgroup was 88.9% female). All acute–chronic comparisons are exploratory and cross-sectional; the small acute sample size, gender imbalance, and absence of within-subject longitudinal follow-up preclude any inference about recovery trajectories.

### BIVSS total scores by TBI phase

**FIGURE 2:**
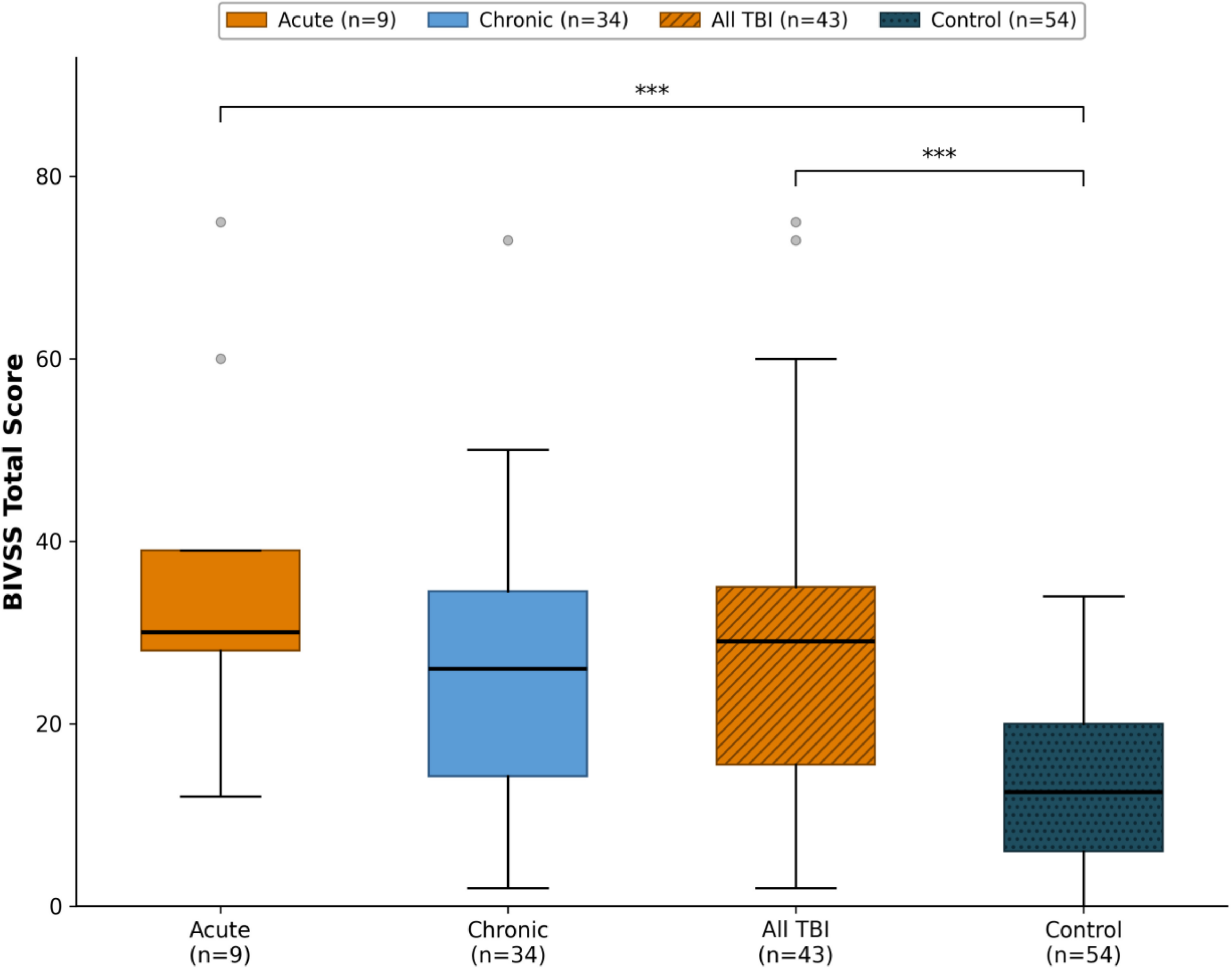
Distribution of BIVSS total scores by TBI phase and control group. Box plots showing the distribution of total BIVSS scores among acute TBI (≤30 days post-injury; ***n*** = 9), chronic TBI (>30 days post-injury; ***n*** = 34), all TBI participants (***n*** = 43), and healthy controls (***n*** = 54). The horizontal line within each box represents the median, box edges represent the interquartile range (IQR), and whiskers extend to 1.5 × IQR; values outside the whiskers are shown as softened gray markers (no data were excluded from any analysis). Group fills/patterns, also given in the legend at the top of the panel, are: Acute = solid orange, Chronic = solid blue, All TBI = orange with diagonal hatching (composite of Acute **+** Chronic), and Control = deep teal with dotted texture; the same color/hatch scheme is used in Figure 3. Significance brackets compare Acute vs. Control and All TBI vs. Control (Welch ***t***-test) using the three-tier convention used throughout: *p < 0.05, **p < 0.01, ***p < 0.001.

Both acute and chronic TBI subgroups demonstrated significantly higher BIVSS total scores than healthy controls (Figure 2). The acute TBI group exhibited the highest mean total score (*M* = 36.3, SD = 19.6), followed by the chronic TBI group (*M* = 25.7, SD = 15.3), with controls showing the lowest scores (*M* = 13.1, SD = 9.0). The difference between acute TBI and controls was statistically significant (*t* = 3.50, *p* = 0.007, Cohen’s *d* = 2.12), as was the difference between chronic TBI and controls (*t* = 4.37, *p* < 0.001, Cohen’s *d* = 1.07). However, the direct comparison between the acute and chronic subgroups did not reach statistical significance (*t* = 1.51, *p* = 0.161, Cohen’s *d* = 0.66), likely due to the small acute sample size and high within-group variability. The cross-sectional design and small acute sample size mean that these data cannot establish whether the higher acute scores reflect a true acute–chronic difference, sampling bias, or both.

### Domain-level symptom patterns

**FIGURE 3:**
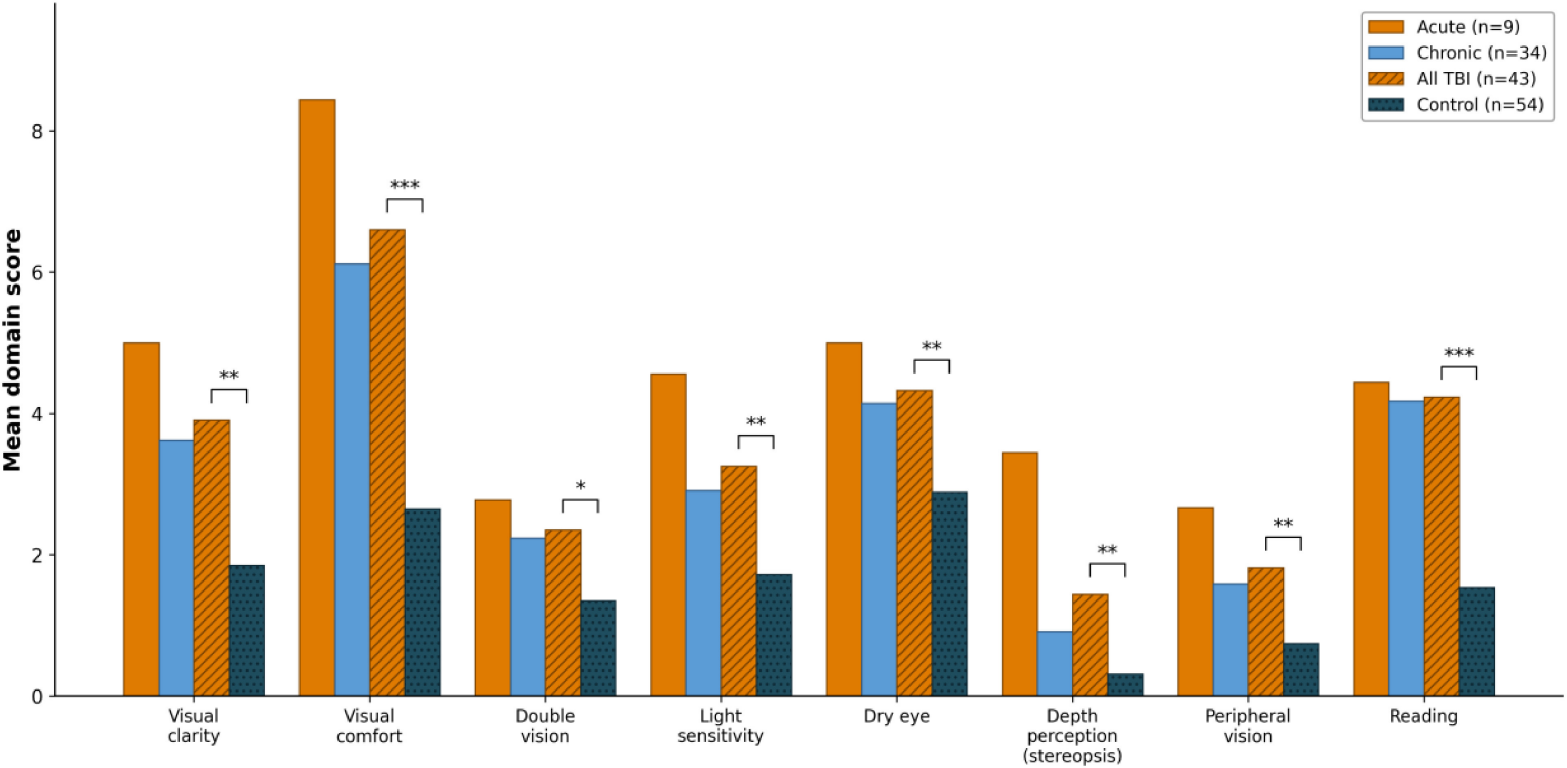
Mean BIVSS domain scores by TBI phase and control group. Comparison of mean scores across eight symptom domains of the Brain Injury Vision Symptom Survey (BIVSS) among acute TBI (≤30 days post-injury; *n* = 9), chronic TBI (>30 days post-injury; *n* = 34), all TBI participants (*n* = 43), and healthy controls (*n* = 54). Note that the *y*-axis shows the mean score within each individual domain (each comprising 2–5 items, scored 0–4); domain means therefore have a much smaller maximum than the total BIVSS score shown in Figure 2 (range 0–112). Group fills/patterns shown in the legend are: Acute = solid orange, Chronic = solid blue, All TBI = orange with diagonal hatching, and Control = deep teal with dotted texture. Significance brackets indicate comparisons between the All TBI and Control groups. *p < 0.05, **p < 0.01, ***p < 0.001.

**FIGURE 4:**
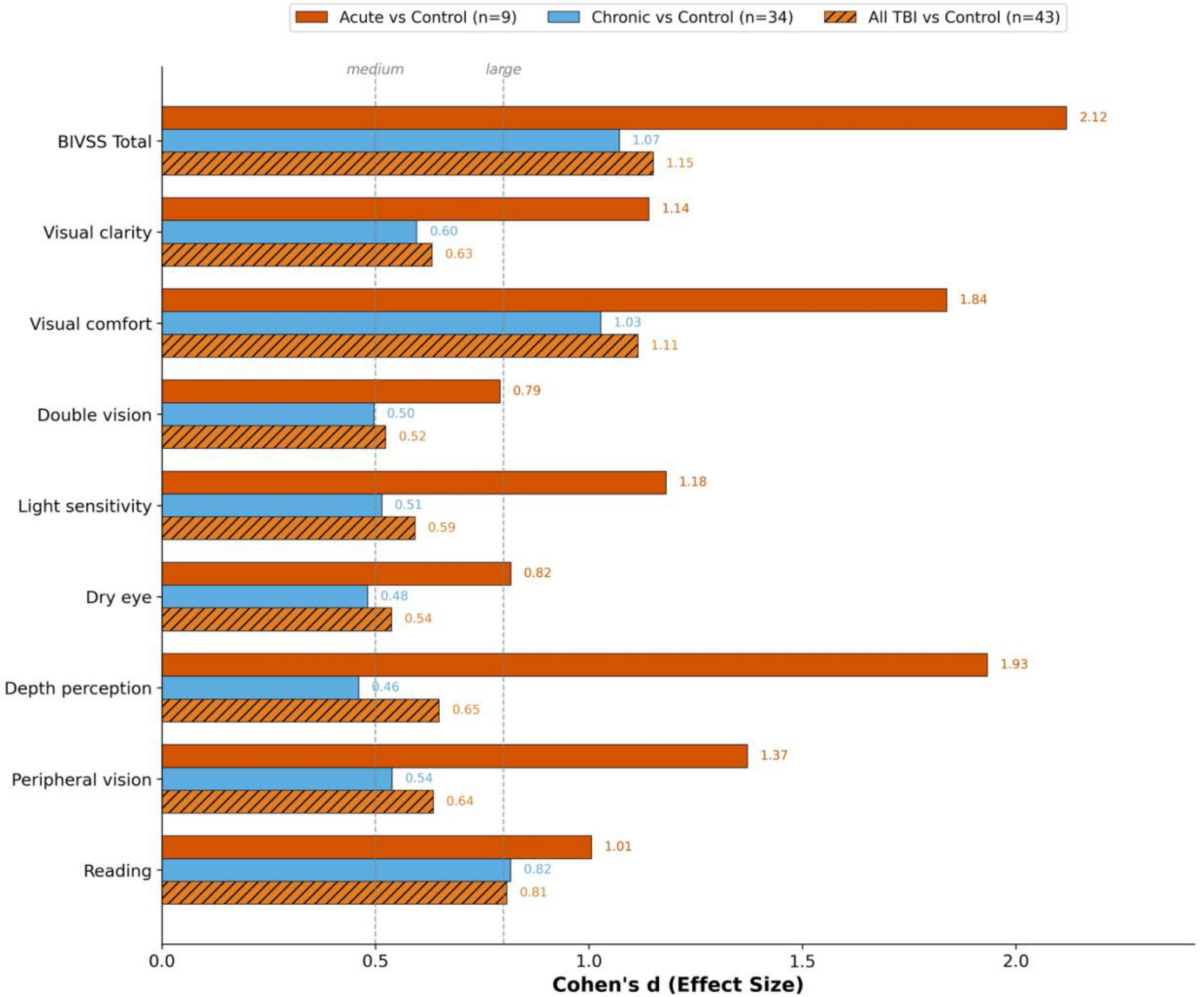
Effect sizes (Cohen’s d) for BIVSS total and domain scores by TBI phase. Horizontal bar chart comparing effect sizes for differences between acute TBI vs. control (*n* = 9), chronic TBI vs. control (*n* = 34), and all TBI vs. control (*n* = 43) across the BIVSS total score and eight symptom domains. Dashed vertical lines indicate thresholds for medium (*d* = 0.5) and large (*d* = 0.8) effect sizes. Notably, depth perception showed the largest cross-sectional difference between the acute and chronic subgroups (*d* = 1.93 vs. *d* = 0.46); because the comparison is between independent subgroups rather than within individuals, this finding is hypothesis-generating and cannot be interpreted as evidence of recovery over time.

Analysis of individual symptom domains revealed distinct cross-sectional patterns between the acute and chronic subgroups (Figure 3). Whereas Figure 2 plots total BIVSS scores (range 0–112), Figure 3 plots the mean score within each individual domain; because each domain comprises only 2–5 items (each scored 0–4), the *y*-axis for Figure 3 is therefore much smaller than for the total score in Figure 2. The most pronounced difference was observed in Depth Perception (Stereopsis), where the acute group reported substantially higher symptom levels (*M* = 3.44) than the chronic group (*M* = 0.91). This was reflected in the effect size comparison, which showed a very large effect for the acute group (*d* = 1.93) but only a moderate effect for the chronic group (*d* = 0.46) when compared to controls (Figure 4). Because acute and chronic data come from different individuals, this difference is hypothesis-generating rather than evidence of within-person recovery; longitudinal studies are required to determine whether stereopsis-related symptoms genuinely diminish over time. In contrast, domains such as Reading and Double Vision showed more persistent symptom burden across both subgroups, with similar effect sizes in acute and chronic strata.

## Discussion

### Principal findings

This study demonstrates that adults with traumatic brain injury (TBI) experience a substantially greater burden of visual symptoms than non-injured controls, as measured by the Brain Injury Vision Symptom Survey (BIVSS). The difference in total BIVSS scores between groups was large (Cohen’s *d* = 1.15), indicating a clinically meaningful effect rather than a marginal statistical difference. Importantly, all eight BIVSS symptom domains were significantly elevated in the TBI group, with the largest effects observed in visual comfort, reading, depth perception, and visual clarity. These findings underscore the pervasive and multidimensional nature of visual dysfunction following TBI and support the use of standardized symptom profiling to flag patients who may benefit from targeted visual assessment after TBI. The BIVSS quantifies symptom burden; objective binocular, accommodative, and oculomotor testing is required to confirm the underlying dysfunctions.

### Clinical interpretation of domain-specific symptom patterns

#### Visual comfort and reading difficulties

The largest between-group differences were observed in the visual comfort and reading domains. These domains capture symptoms such as eye strain, visual fatigue, headaches during near tasks, difficulty sustaining reading, and losing one’s place on the page. Clinically, this symptom constellation is highly consistent with convergence insufficiency and accommodative dysfunction, which are among the most frequently reported visual sequelae of TBI (Almutairi et al., 2025b,c; Brahm et al., 2009; Capó-Aponte et al., 2012; Ciuffreda et al., 2008; Green et al., 2010a). A systematic review and meta-analysis reported a pooled prevalence of accommodative dysfunction after TBI of 42.8% of individuals after TBI, while convergence insufficiency has been reported in approximately 36% of individuals following TBI (Merezhinskaya et al., 2019). Post-TBI accommodative and binocular vision dysfunctions often present as reading-related visual fatigue, asthenopia, and intermittent blur or diplopia rather than constant diplopia; therefore, patients may report substantial visual disability despite normal distance visual acuity. Symptom-based tools, such as the Brain Injury Vision Symptom Survey (BIVSS), can help quantify this burden and identify patients who require targeted accommodative and binocular vision assessment (Laukkanen et al., 2017; Magone et al., 2014; Merezhinskaya et al., 2019).

### Visual clarity and depth perception

Elevated scores in the visual clarity domain reflect symptoms such as fluctuating blur and difficulty shifting focus between distances. These complaints are frequently linked to instability of the accommodative system and impaired accommodative dynamics following TBI (Almutairi et al., 2025c; Almutairi, 2025; Ciuffreda and Thiagarajan, 2022; Dutta et al., 2024; Green et al., 2010b; Haensel et al., 2024). Disruption of cortical and subcortical networks involved in accommodation control can result in delayed or inaccurate focusing responses, contributing to intermittent blur even in the presence of normal refractive correction (Green et al., 2010a).

The depth perception (stereopsis) domain also showed a moderate-to-large effect size. Difficulties judging distance, negotiating steps, or perceiving spatial relationships have been widely reported in TBI populations and are often associated with binocular coordination deficits and dorsal visual stream involvement (Miller et al., 1999; Padula et al., 2017). These symptoms have important functional implications, as impaired stereopsis has been linked to increased fall risk, reduced mobility confidence, and challenges with activities such as driving and sports participation (Dhital et al., 2010; Moyegbone et al., 2025).

### Light sensitivity, peripheral vision, and sensory integration

Symptoms related to light sensitivity and peripheral vision were also significantly elevated in the TBI group. Photophobia is a well-recognized post-TBI symptom and is thought to reflect altered cortical excitability and disrupted sensory filtering mechanisms within thalamocortical pathways (Diel et al., 2021; Mares et al., 2019). Similarly, peripheral visual complaints may reflect impairments in visual-vestibular integration and attentional processing rather than primary retinal pathology (Alnawmasi et al., 2022; Wallace and Lifshitz, 2016).

These symptoms are particularly relevant in real-world environments that involve complex visual stimuli, such as busy clinics, workplaces, or public spaces. Even moderate elevations in these domains may therefore contribute disproportionately to functional disability and reduced quality of life after TBI (Brahm et al., 2009; Rauchman et al., 2023).

### Double vision: less prominent but clinically relevant

Although the double vision domain demonstrated the smallest effect size, it remained significantly elevated in the TBI group. This finding aligns with previous reports indicating that persistent, constant diplopia is less common than more subtle binocular instability following TBI (Fowler et al., 1996; Master et al., 2022). Many patients experience intermittent or task-dependent diplopia that emerges during fatigue, sustained near work, or visually demanding activities, which may not be captured during brief clinical assessments. Importantly, even moderate diplopia scores should prompt clinicians to evaluate ocular alignment, fusional reserves, and vergence stability, as these patients may benefit from targeted interventions such as prism correction or vision rehabilitation (Smaakjær et al., 2022; Watabe et al., 2019).

### Comparison with previous literature

The present findings are consistent with a growing body of literature documenting the high prevalence and clinical impact of visual dysfunction after TBI. Prior studies have often focused on single visual diagnoses or specific subpopulations (e.g., sports-related concussion, military cohorts), whereas the current study extends this work by demonstrating broad symptom elevation across multiple functional domains within a clinically heterogeneous adult sample. Notably, most previous investigations have emphasized prevalence estimates or diagnostic frequencies, whereas our study highlights the magnitude of symptom burden using standardized effect sizes. This approach provides additional clinical insight by quantifying the extent to which visual symptoms differentiate individuals with TBI from uninjured controls.

### Potential clinical utility of the BIVSS

The BIVSS offers several advantages for clinical practice. It is brief, easy to administer, and captures symptoms that are directly relevant to patients’ daily visual experiences. As emphasized by Laukkanen et al. (2017), the BIVSS is a symptom-profiling and screening-prompt instrument and is not intended as a stand-alone diagnostic test for any specific binocular, accommodative, or oculomotor disorder. The observed ROC performance in this case–control sample (AUC = 0.79) suggests that, pending external validation in clinically representative populations, the BIVSS total score may discriminate between individuals with and without TBI-related visual symptomatology.

Importantly, the candidate cut-off values reported here (e.g., ≥12, ≥22, and ≥28 points) are exploratory anchor points derived from a single, modestly sized case–control sample without external validation. The maximum-Youden cut-off of ≥28 points achieved high specificity (96.3%) but low sensitivity (55.8%), and the AUC of 0.79 is moderate; these values should not be interpreted as clinically validated screening thresholds for the Arabic BIVSS. Because the case–control sampling design fixes the apparent prevalence of TBI at approximately 44%, the corresponding positive and negative predictive values reflect that sampling ratio rather than clinical prevalence and are not directly generalizable to clinical populations. External validation in independent prospective samples is required before any specific cut-off can be recommended for clinical use. Beyond the total score, domain-level profiles may help guide which targeted assessments to consider next, for example vergence and accommodation testing in patients with high visual comfort and reading scores, or stereopsis and balance evaluation in those with elevated depth perception symptoms. Because objective neuro-optometric testing was not performed in the present study, the links between BIVSS domain scores and specific binocular, accommodative, or oculomotor disorders remain inferential and require confirmation in studies that pair the BIVSS with objective measures (Rakoczy, 2015).

### Strengths and limitations

Strengths of this study include the use of clinically confirmed TBI diagnoses and the recruitment of a non-injured control group from the same geographic region, screened to exclude neurological or ocular conditions affecting vision. The emphasis on standardized effect sizes and domain-specific symptom patterns enhances the clinical interpretability of the findings. Several limitations should be acknowledged. First, the sample size is modest (43 TBI, 54 controls) and was recruited by convenience from a single region, so the findings should be regarded as exploratory and may not generalize to other populations. In particular, the TBI cases were clinic-recruited from neurology, rehabilitation, and general optometry settings, whereas the control group was drawn from the Qassim University student body; these groups may not be fully comparable in age structure, healthcare-seeking behavior, or baseline symptom reporting, and this mismatch may inflate the apparent between-group differences (effect sizes, AUC, sensitivity, and specificity) reported here. Second, the TBI cohort is highly heterogeneous: time since injury ranged from 1 to 9490 days (median 730 days), formal severity classification was unavailable for 10 of 43 participants, and the acute subgroup (*n* = 9) was small and unbalanced for gender. This heterogeneity limits the interpretability of the pooled TBI mean, of the exploratory acute–chronic comparison, and of the candidate score cut-offs, and should be considered alongside the descriptive findings. Third, this analysis adds local Arabic case–control data but does not establish Arabic-specific measurement behavior: cross-language measurement invariance, differential item functioning, factor structure, and clinically validated screening thresholds were not assessed and are not claimed for the Arabic BIVSS on the basis of these data; full psychometric work is reported in the companion paper. Fourth, because of the case–control sampling design, the AUC, sensitivity, specificity, and especially the positive and negative predictive values reported here are sample-specific estimates and require external validation in independent prospective samples before any cut-off can be recommended for clinical use. Fifth, the cross-sectional design precludes causal inference and prevents any inference about within-person recovery from the acute–chronic comparison, and symptom reports were not paired with objective visual performance measures, so links between specific BIVSS domains and underlying accommodative, binocular, or oculomotor dysfunction remain inferential. Future studies integrating symptom surveys with detailed neuro-optometric testing, well-characterized severity strata, and longitudinal follow-up will be essential to further elucidate the mechanisms underlying these symptom patterns, to confirm or refute the apparent acute–chronic differences, and to externally validate any candidate Arabic BIVSS screening cut-offs in clinically representative populations.

## Data Availability

The original contributions presented in this study are included in this article/Supplementary material, further inquiries can be directed to the corresponding author.
