# Supplementary material for: Visual symptom burden after traumatic brain injury: a case-control evaluation of the Arabic BIVSS
Source: Front Neurosci. 2026 May 28;20:1827721. doi: 10.3389/fnins.2026.1827721 (PMC13253801; doi:10.3389/fnins.2026.1827721)
Supplement: Supplementary file 1 [file Table_1.docx]

**Supplementary Table 1:**

Diagnostic performance of Arabic-BIVSS total score cutoffs for identifying TBI-associated visual symptoms. Values represent sensitivity, specificity, positive predictive value (PPV), negative predictive value (NPV), Youden's J index, and overall accuracy at each threshold. The optimal cutoff (≥28 points) was determined by maximizing Youden's J.

| Cutoff | Sensitivity | Specificity | PPV | NPV | Youden's J | Accuracy |
| --- | --- | --- | --- | --- | --- | --- |
| ≥10 | 88.4% | 40.7% | 54.3% | 81.5% | 0.291 | 61.9% |
| ≥12 | 83.7% | 46.3% | 55.4% | 78.1% | 0.300 | 62.9% |
| ≥14 | 79.1% | 53.7% | 57.6% | 76.3% | 0.328 | 64.9% |
| ≥16 | 74.4% | 61.1% | 60.4% | 75.0% | 0.355 | 67.0% |
| ≥18 | 67.4% | 68.5% | 63.0% | 72.5% | 0.360 | 68.0% |
| ≥20 | 67.4% | 70.4% | 64.4% | 73.1% | 0.378 | 69.1% |
| **≥22** | **65.1%** | **81.5%** | **73.7%** | **74.6%** | **0.466** | **74.2%** |
| ≥24 | 58.1% | 83.3% | 73.5% | 71.4% | 0.415 | 72.2% |
| ≥26 | 55.8% | 92.6% | 85.7% | 72.5% | 0.484 | 76.3% |
| **≥28** | **55.8%** | **96.3%** | **92.3%** | **73.2%** | **0.521** | **78.4%** |
| ≥30 | 44.2% | 96.3% | 90.5% | 68.4% | 0.405 | 73.2% |
| ≥31 | 39.5% | 96.3% | 89.5% | 66.7% | 0.358 | 71.1% |
