# Supplementary material for: Visual symptom burden after traumatic brain injury: a case-control evaluation of the Arabic BIVSS
Source: Front Neurosci. 2026 May 28;20:1827721. doi: 10.3389/fnins.2026.1827721 (PMC13253801; doi:10.3389/fnins.2026.1827721)
Supplement: Supplementary file 2 [file Table_2.docx]

**Supplementary Table 2:**

Demographic and clinical characteristics of the acute TBI, chronic TBI, and control subgroups. Values are presented as mean ± SD for continuous variables and n (%) for categorical variables. Acute TBI is defined as ≤30 days post-injury; chronic TBI is defined as >30 days post-injury.

| Characteristic | Acute TBI (n=9) | Chronic TBI (n=34) | Control (n=54) | p-value |
| --- | --- | --- | --- | --- |
| **Age (years), mean ± SD** | 21.3 ± 2.3 | 28.6 ± 12.5 | 25.0 ± 6.8 | 0.059ᵃ |
| **Gender, n (%)** |  |  |  | 0.042ᵇ |
| Female | 8 (88.9%) | 19 (55.9%) | 24 (44.4%) |  |
| Male | 1 (11.1%) | 15 (44.1%) | 30 (55.6%) |  |
| **Time since injury** |  |  |  |  |
| Mean ± SD (days) | 11.4 ± 10.8 | 2,469.2 ± 2,542.4 | N/A |  |
| Median (IQR) (days) |  | 1278 (365–4471) | N/A |  |
| Range (days) | 1–30 | 90–9,490 | N/A |  |
| **TBI Severity, n (%)** |  |  |  |  |
| Mild | 4 (44.4%) | 7 (20.6%) | N/A |  |
| Moderate | 1 (11.1%) | 9 (26.5%) | N/A |  |
| Severe | 0 (0.0%) | 3 (8.8%) | N/A |  |
| Emergency | 1 (11.1%) | 8 (23.5%) | N/A |  |
| Missing | 3 (33.3%) | 7 (20.6%) | N/A |  |
| **BIVSS Total Score, mean ± SD** | 36.3 ± 19.6 | 25.7 ± 15.3 | 13.1 ± 9.0 | <0.001ᵃ |

ᵃ One-way ANOVA
ᵇ Chi-square test
